# Cephalometric features associated with the mentolabial angle and lower lip eversion in young adults: A cross-sectional study

**DOI:** 10.4317/jced.63016

**Published:** 2025-08-01

**Authors:** Luis Ernesto Arriola-Guillén, André Alexis Díaz-Quevedo, Yalil Augusto Rodríguez-Cárdenas, Gustavo Armando Ruíz-Mora, Heraldo Luis Días-Da Silveira

**Affiliations:** 1 Ph.D. and Associate Professor of the Division of Orthodontics, Universidad Científica del Sur, Lima, Perú; 2 Dentist, School of Dentistry, Universidad Científica del Sur, Lima, Perú; 3 DDS, MSc, PhD. Associate Professor of the Division of Oral and Maxillofacial Radiology, School of Dentistry, Universidad Nacional de Colombia, Bogotá, Colombia; 4 DDS, MSc, PhD. Associate Professor of the Division of Orthodontics, Faculty of Dentistry, Universidad Nacional de Colombia, Bogotá D.C, Colombia; 5 DDS, MSc, PhD. Associate Professor of Department of Oral Surgery and Orthopedics, Division of Dental Radiology, Dental School, Federal University of Rio Grande do Sul (UFRGS), Porto Alegre, Rio Grande do Sul, Brazil

## Abstract

**Background:**

Understanding the cephalometric factors that affect the mentolabial angle and lower lip eversion is essential for orthodontics. The objective was to evaluate the influence of various skeletal and dentoalveolar cephalometric features associated with the mentolabial angle and lower lip eversion in young adults.

**Material and Methods:**

This cross-sectional study assessed lateral head radiographs of individuals between the ages of 15 and 40 years. Two trained and calibrated evaluators performed angular and linear cephalometric measurements utilizing BlueSky Plan 4 software. The variables evaluated in this study included the presence of the mentolabial angle, labial eversion, overjet, and the position and inclination of the lower central incisors (measured by I-NB, I.NB, IMPA) as well as the upper central incisors (measured by I-NA, I.NA, UIPP). Additionally, we assessed the sagittal positions of the maxilla and mandible using SNA and SNB measurements, along with the sagittal and vertical skeletal relationships determined by the ANB and FMA angles. We applied multiple linear and binary logistic regression tests for statistical analysis (*p*<0.05).

**Results:**

138 radiographs were evaluated (73 females and 65 males). The mentolabial angle is, on average, 7.68° greater in women than men (*p*=0.001). An increase in the IMPA and overjet decreases 0.40° (*p*=0.012) and 2.02° (*p*=0.003) in the mentolabial angle, respectively. Likewise, females are 82% less likely to experience lip eversion than males (exp B = 0.18, 0.05 to 0.60 CI 95%; *p*=0.006). Furthermore, for each degree increase in lower incisor inclination (I. NB) or overjet, the risk of developing lip eversion increases by 1.17 times (1.02 - 1.34 CI to 95%, *p*=0.023) and 1.85 times (1.23 - 2.78 CI to 95%, *p*=0.003), respectively.

**Conclusions:**

The inclination of the lower incisors and the overjet primarily influences the mento-labial angle. Women tend to have a greater mento labial angle, meaning it is more retrusive, compared to men. Additionally, the likelihood of lip eversion is higher in males. For each degree of increased lower incisor inclination or overjet, the risk of developing lip eversion also rises.

** Key words:**Cephalometry, Chin, Dental Esthetics, Lip.

## Introduction

Lower lip eversion is defined as an improper resting position of the lower lip, characterized by a forward fold that creates a pronounced mento labial groove. Depending on its severity, this condition can lead to increased visibility of the labial mucosa. This condition is often perceived negatively regarding facial aesthetics [1-3]. The appearance of labial eversion has been associated with various factors, including positive overjet, the inclination of the lower and upper central incisors, and the position of the mandible, among other cephalometric and muscular characteristics [4]. Although these associations exist, the degree to which each factor impacts the eversion is unclear. Gaining insight into these influences could benefit dental practice.

Likewise, the mentolabial angle is a cephalometric measurement that quantifies the protrusion of the lower lip in relation to the soft chin [5-7]. A smaller mentolabial angle indicates a greater protrusion of the lower lip, [8] and its impact on facial aesthetics has been assessed in various studies [9-12]. Research suggests that this angle is one of the key factors associated with facial attractiveness [9,10]. Specifically, one study found that mentolabial angles between 107° and 118° are generally considered more attractive, while angles outside this range are perceived as less attractive [11]. Additionally, Parul *et al*. [12] concluded that the depth of the mento labial sulcus contributes to the overall attractiveness of Class II and III facial types. However, no studies have quantified the impact of specific factors, such as incisor positions, inclinations, overjet, and bony structures like skeletal relationships, which could influence and modify the mentolabial angle. Gaining insight into which structures most significantly affect lower lip eversion and the mento labial angle will enable clinicians to prioritize correcting these factors in their treatment plans, benefiting patients by achieving better outcomes.

For these reasons, this study aims to evaluate the influence of various skeletal and dentoalveolar cephalometric features associated with the mentolabial angle and lower lip eversion in young adults, seeking to determine which factors most significantly affect the position of the lower lip and, consequently, facial aesthetics. Additionally, the information presented in this article may be valuable for understanding the growing demand from patients seeking facial contouring procedures alongside orthodontic treatments [13]. By understanding the modifications of the mentolabial angle and labial eversion, professionals can better educate their patients and enhance their knowledge when addressing related conditions.

## Material and Methods

This retrospective, cross-sectional study followed the guidelines of the STROBE checklist and the Declaration of Helsinki. It used the database of a previous study approved by the Ethics Committee of the Federal University of Rio Grande do Sul, Porto Alegre, Brazil, with protocol registration number 1.890.015.

A PEO strategy was proposed for sample selection, which included the following information: Population: Young adults with different Angle malocclusions. Exposition: Individuals with different skeletal and dentoalveolar cephalometric values. Outcomes: Mentolabial angle and lower lip eversion. The sample size was calculated using Openepi.com (https://www.openepi.com/SampleSize/SSPropor.htm) with data obtained from a previous pilot test. A formula was applied to estimate a proportion, and the following information was recorded: percentage influence of the selected cephalometric predictor variables evaluated on the development of lower lip eversion (R2 Cox y Snell = 10%), confidence level (95%), test power (80%), and precision (5%). The minimum required sample size was 138 lateral head radiographs. To achieve this number, 198 X-rays were analyzed, with the sole reason for excluding the others being that they did not meet the selection criteria.

Inclusion criteria included successive lateral head radiographs of individuals aged 15 to 40 years, of both sexes, conducted between January and March 2023, who attended a private radiology department in Porto Alegre, Brazil, and who gave their approval for the use of their radiographs for research purposes ( Table 1). Lateral radiographs with poor clarity, those of individuals with syndromic craniofacial deformities, those undergoing orthopedic or orthodontic treatment with braces or surgical plates, those with tooth loss, supernumerary teeth, cranial deformities, or head malformations, and patients with dentoalveolar trauma, were excluded.

- Training and Calibration

Two trained and calibrated observers conducted all measurements: an orthodontist with over 10 years of experience in cephalometric and an experienced dentist. Fifty radiographs were taken at two different times, spaced two weeks apart, to obtain calibration values. The intraclass correlation coefficient was used for quantitative variables, while the kappa statistic was employed for qualitative variables. Calibration values needed to exceed 0.8 for both tests and the measurement error had to be less than one unit.

- Measurement of Variables

**Figure 1:**
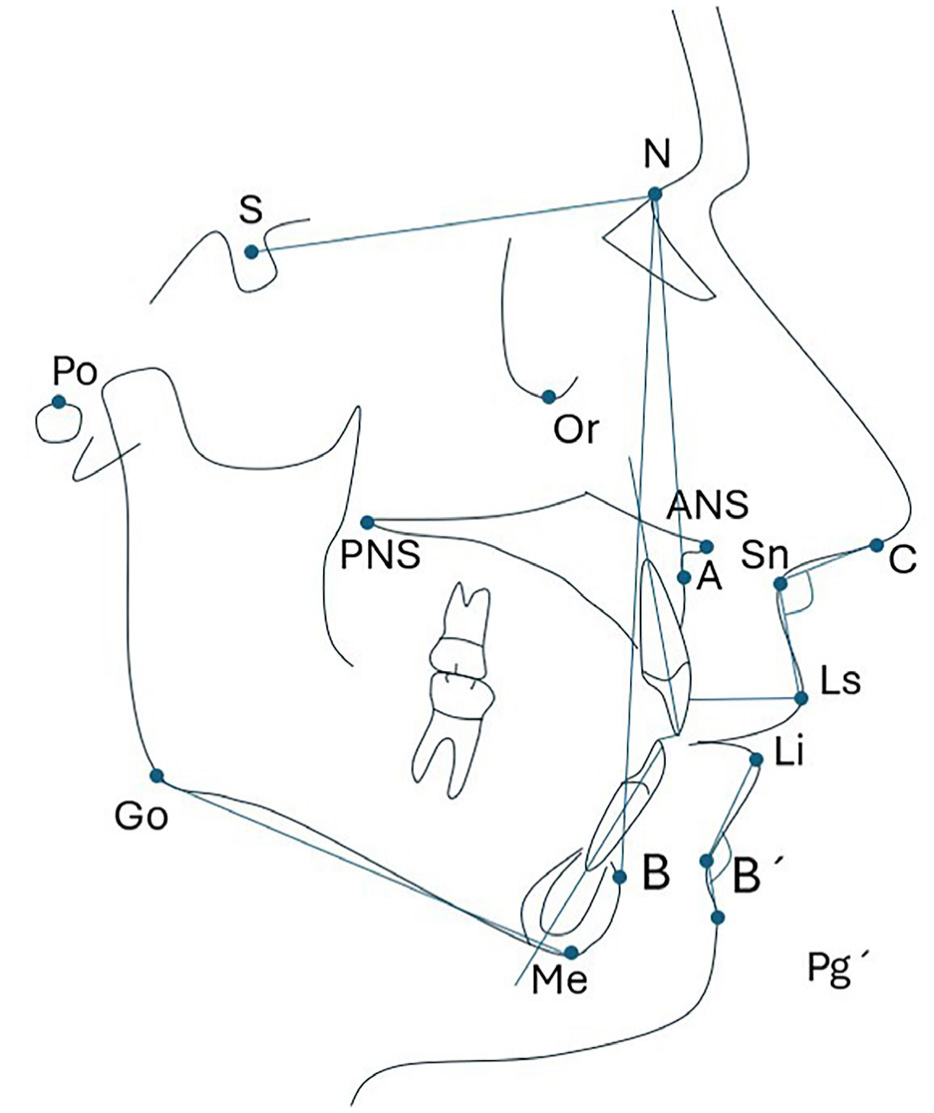
Illustration of the cephalometric characteristics assessed in the sample studied.

All cephalometric measurements were conducted using the BlueSky Plan 4 cephalometric analysis software (USA). The lateral head radiographs were analyzed in JPG format. The following angular and linear cephalometric measurements were evaluated: the presence or absence of labial eversion, mentolabial angle, overjet, and the inclinations of the lower (I-NB, I. NB, IMPA) and upper (I-NA, I.NA, UIPP) central incisors. Additionally, the positions of the maxilla (SNA) and mandible (SNB) were assessed, along with the sagittal and vertical skeletal relationship angles (ANB, FMA) ( Table 2, Fig. 1).

- Statistical analysis

Statistical analysis was performed using SPSS version 24 (NY, USA). Cephalometric variables were initially described and then evaluated using the Shapiro-Wilk test to assess the normality of the data. Multiple linear regression was conducted to analyze the mento labial angle, while binary logistic regression was used to evaluate the presence of labial eversion. The “overfit” method was applied for multiple regression analysis, allowing for two consecutive regressions. In the first regression, variables with a *p-value* < 0.20 were identified, followed by a second regression that included only those selected variables. The significance level of *p* < 0.05 was established.

## Results

 Table 3 presents the results of a multiple linear regression analysis assessing the impact of predictor variables on the mentolabial angle. This analysis focused on variables that showed a *p-value* of less than 0.2 in a preliminary regression. The findings indicate that the mentolabial angle is, on average, 7.68° higher in women compared to men (*p* = 0.001). Additionally, for each degree increase in the Incisor Mandibular Plane Angle (IMPA), the mentolabial angle decreases by 0.40° (*p* = 0.012). Furthermore, for each millimeter increase in overjet, the mentolabial angle decreases by 2.02° (*p* = 0.003).  Table 4 displays the results of a binary logistic regression analyzing the influence of various predictor variables on the presence of lip eversion. This analysis focused on variables with a *p-value* of less than 0.200 in a preliminary regression using the overfit method. The results show that females are 82% less likely to experience lip eversion than males (B exp. = 0.18, 0.05 to 0.60 CI 95%; *p*=0.006). Additionally, for each degree that the lower incisor inclination (I. NB) increases, the risk of developing lip eversion rises by 1.17 times (95% CI: 1.02–1.34, *p* = 0.023). Furthermore, for every millimeter increase in overjet, the risk of developing lip eversion rises by 1.85 times (95% CI: 1.23–2.78, *p* = 0.003).

## Discussion

Understanding the numerous factors that influence the mentolabial angle and lower lip eversion is essential for an effective orthodontic practice. As patients increasingly prioritize orofacial harmony during orthodontic treatment, orthodontists need to educate them about these key elements. By comprehending how these anatomical components interact and contribute to the overall aesthetic of the face, patients can develop real expectations and a deeper appreciation for the treatment process. This understanding can lead to a more satisfying outcome. The mentolabial angle is a cephalometric measurement that assesses the inclination of the lower lip about the soft chin. Orthodontists can determine the harmony of facial features by analyzing a lateral view of the profile, as the position of the lower lip is crucial for the aesthetics of this area [13]. Previous studies have identified the position and inclination of the incisors and the maxillary bone base as the main factors influencing this angle [9-12]. However, it is crucial to validate this relationship across various samples and to identify the specific contribution of each factor that has not been thoroughly examined using suitable statistical methods, particularly regression analysis. Furthermore, the eversion of the lower lip significantly impacts the harmony of the facial profile, and the placement of specific anatomical structures affects its overall appearance. Nonetheless, the exact influence of each structure remains unclear, making this information valuable for clinical orthodontic practice. Therefore, this research aims to evaluate the impact of cephalometric characteristics on lower lip eversion and variations in the mentolabial angle among young adults.

Previous studies have indicated that variations in the mentolabial angle are primarily influenced by the position and angulation of the incisors. However, most of these studies have mainly focused on correlations, with limited analysis of the weight each factor contributes to this issue. Understanding these results could be valuable when planning orthodontic treatments aimed at improving profiles with labial eversion. Additionally, some studies suggest that the correlation between upper and lower soft tissue and hard tissue variables is reliable for certain variables but not consistent for all [14,15]. Therefore, more research should be conducted to evaluate these influences.

Our research examined the influence of key cephalometric variables typically used in cephalometric analyses in orthodontics. Thus, we evaluated the skeletal relationships, the position of the maxillary bases, the position and inclination of the incisors, and demographic characteristics, trying to identify the variables with the most significant impact on mentolabial angle and lip eversion appearance. Thus, we employed the overfit method for regression. This approach entails performing two regression analyses to pinpoint the most influential variables, culminating in an additional regression to evaluate the significance of these critical factors. Thus, some variables, such as vertical and sagittal skeletal patterns and jaw positions, did not significantly influence the mento-labial angle, unlike other facial features [16]. Although for some studies, the ANB was mainly influential [17], or the body mass index [18].

The results of the multiple linear regression analysis evaluating predictor variables on changes in the mentolabial angle indicate that, on average, the mentolabial angle is 7.68° higher in women than in men (*p* = 0.001) meaning it is more retrusive, compared to men. This finding is consistent with previous studies that have reported similar results [19,20], advocating for orthodontic treatment since many studies indicate that straighter facial profiles are perceived as more attractive [21-23]. We also discovered that for each degree increase in the inclination of the lower incisors (IMPA), the mentolabial angle decreases by 0.40° (*p* = 0.012). This result indicates that a greater inclination of the lower incisors results in a sharper mentolabial angle, contributing to lip protrusion and ultimately affecting the aesthetic profile. Furthermore, the overjet has a significant influence, as each millimeter increase in overjet corresponds to a decrease of 2.02° in the mentolabial angle (*p* = 0.003). This finding implies that patients with a greater positive overjet will have an increasingly protruded mentolabial angle, leading to noticeable aesthetic alterations.

Likewise, when we evaluate the influence of predictor variables on lip eversion using a binary logistic regression, we identified that females are 82% less likely to experience lip eversion than male (*p*=0.006). This information may be common, even though our sample consisted entirely of Latin American patients [19,20]. Furthermore, it may represent a universal aspect that researchers should consider in future studies with different populations. Furthermore, orthodontists must be aware of this aspect when treating male patients. Besides, for each degree increase in the inclination of the lower incisors (I. NB), the risk of developing lip eversion increases by 1.17 times (95% CI: 1.02–1.34, *p* = 0.023). This finding suggests that a greater inclination of the lower incisors leads to lip protrusion, ultimately affecting the aesthetic profile. Additionally, for every millimeter increase in overjet, the risk of developing lip eversion rises by 1.85 times (95% CI: 1.23–2.78, *p* = 0.003). This aspect occurs because, when there is space between the incisal edges, the upper portion of the lower lip moves forward into this gap, resulting in labial eversion. Therefore, cases with a larger overjet will experience more labial eversion, and orthodontists should consider this when planning their treatments.

This research highlights the significance of the most commonly used cephalometric variables in orthodontics, particularly their influence on the mentolabial angle and lower lip eversion. While additional variables could have been considered, the focus was placed on those most frequently used in clinical practice. Moreover, our results primarily demonstrate internal validity; therefore, further studies in other populations are necessary. Ultimately, this information will assist orthodontists in planning treatment for patients who need adjustments to the mentolabial angle or have issues with lower lip eversion.

## Conclusions

The mentolabial angle is primarily influenced by the lower incisor angle and the overjet. Typically, this angle appears straighter in women than in men. Furthermore, the risk of lip eversion is lower in females; with each degree increase in the inclination of the lower incisors or the overjet, the probability of developing lip eversion rises. This information will assist orthodontists in effectively planning their treatments to modify the mentolabial angle.

## Figures and Tables

**Table 1:** Demographic characteristics of the sample (n=138).

Sex	n	Mean	SD	p
Male	65	29.90	6.49	0.354
Female	73	28.90	4.85	

Student T test

**Table 2:** Description of cephalometric features measured.

Value	Cephalometric definition
Mentolabial angle	Value of the mentolabial angle obtained from the lines soft pogonion-mentolabial sulcus, mentolabial sulcus-lower lip.
SNA	Angular value formed between point Sella, Nasion, point A.
SNB	Angular value formed between point Sella, Nasion, point B.
ANB	Angular value formed between point A, Nasion, point B.
FMA	Angular value formed between Frankfort plane with mandibular plane.
I-NB	Distance in millimeters from the incisal edge of the lower central incisor to the NB line.
INB	Angle formed between the NB line and the longitudinal axis of the lower central incisor.
IMPA	Angle formed between the mandibular plane (Go-Me) and the longitudinal axis of the lower central incisor.
I-NA	Distance in millimeters from the incisal edge of the superior central incisor to the NA line.
INA	Angle formed between the NA line and the longitudinal axis of the superior central incisor.
UIPP	Angle formed between the palatal plane (ANS-PNS) and the longitudinal axis of the upper central incisor.
Overjet	Distance in millimeters from the incisal edge of the upper central incisor to the incisal edge of the lower central incisor.

**Table 3:** Second multiple linear regression to evaluate the influence of predictor variables on the mentolabial angle.

Variables	B	p	95.0% confidence interval for B	
			Lower limit	Upper limit
Constant	185.22	<0.001*	133.91	236.53
Male	---	---	---	---
Female	7.68	0.001*	3.08	12.27
IMPA	-0.40	0.012*	-0.71	-0.08
I.NA	0.26	0.298	-0.23	0.75
UIPP	-0.16	0.487	-0.63	0.30
Overjet	-2.02	0.003*	-3.35	-0.68

* Significant, R2 =18.5%

**Table 4:** Binary logistic regression to evaluate the influence of predictor variables on the lip eversion.

Variables	B Exp	p	95.0% confidence interval for B	
			Lower limit	Upper limit
Male	---	---	---	---
Female	0.18	0.006*	0.05	0.60
I.NB	1.17	0.023*	1.02	1.34
IMPA	0.93	0.264	0.83	1.05
I.NA	0.97	0.473	0.89	1.05
Overjet	1.85	0.003*	1.23	2.78
Constant	0.13	0.667		

* Significant, R2 Cox y Snell =16.9%

## Data Availability

The datasets used and/or analyzed during the current study are available from the corresponding author.
